# Can stellate ganglion blockage be an alternative treatment for refractory ventricular arrhythmias: Case series

**DOI:** 10.1097/MD.0000000000034135

**Published:** 2023-06-30

**Authors:** Çağatay Küçükbingöz, Ömer Tepe, Çağlar Özmen, Hayri T. Özbek, Ali Deniz

**Affiliations:** a Ministry of Health Adana City Training & Research Hospital, Department of Anesthesiology & Reanimation, Discipline of Pain Medicine, Adana, Turkey; b Cukurova University Faculty of Medicine, Department of Cardiology, Adana, Turkey; c Osmaniye State Hospital, Department of Cardiology, Adana, Turkey; d Cukurova University Faculty of Medicine, Department of Anesthesiology & Reanimation, Discipline of Pain Medicine, Adana, Turkey.

**Keywords:** stellate ganglion, ultrasound, ventricular arrythmias

## Abstract

Ventricular tachycardia (VT) and ventricular fibrillation (VF) are life-threatening conditions which increase in frequency over the years. Electrical storm (ES) is defined as the occurrence of 3 or more continuous ventricular arrhythmia. The sympathetic nervous system has an important role in Ventricular arrythmias (VA) and is the target of treatment. Studies show that stellate ganglion blockade (SGB) can reduce cardiac sympathetic tone and is an alternative bridge therapy in VAs.

**Patient concerns:**

Among the patients who were admitted to the hospital with the complaints of general condition disorder and palpitation.

**Diagnosis:**

Patients were referred to the Cardiology department and diagnosed VA and ES. Patients who applied to the Cardiology Department with the diagnosis of VA or ES and did not benefit from antiarrhythmic drug therapy were selected and evaluated by a team of 2 anesthesiologists (cardiothoracic and pain specialists) and 2 cardiologists (1 of whom is an electrophysiology specialist).

**Intervention:**

In our study, ultrasound (USG) guided left SGB was applied to 10 VA and ES patients with implantable cardiac defibrillator (ICD). The 6-month results of the patients were evaluated retrospectively. For blockage, the solution was prepared by adding 8 mg dexamethasone, 40 mg lidocaine and 10 mg bupivacaine to 10 mL with physiological saline. The success of the procedure was evaluated with the development of Horner syndrome in the left eye.

**Outcomes:**

Resistant VA developed in 2 of 10 patients who had left SGB due to VF/VT ES and were excluded from the study. One (1) month after the procedure it was seen that there was a statistically significant decrease in the number of shocks in 8 patients in the 6th month controls compared to the pre-procedure. The number of VES in the 1st and 6th months of the patients was also statistically significantly decreased compared to the pre-SSD (*P* = .01, *P* = .01, *P* = .01, respectively).

**Conclusion:**

Unilateral USG-guided SGB application is an effective and safe method in patients with ES and VA. Long-term results can be satisfactory in successful responders in whom SGB is performed with a combination of local anesthetic and steroid.

## 1. Introduction

Ventricular tachycardia (VT) and ventricular fibrillation (VF) are life-threatening conditions which increase in frequency over the years.^[1]^ Ventricular arrhythmias (VA) causes more than 450,000 deaths per year in the USA.^[2]^ Electrical storm (ES) is defined as the occurrence of 3 or more continuous VA episodes over 24 hours.^[3,4]^ Even in the presence of an implantable cardiac defibrillator (ICD), an ES carries an 8 times higher risk of arrhythmic death.^[5]^ Standard therapy in ES and VA is antiarrhythmic drugs and catheter ablation.^[6]^

The sympathetic nervous system has an important role in VA and is the target of treatment.^[7]^ Cardiac sympathetic denervation, surgical resection of the lower half of the stellate (cervicothoracic) ganglion and the sympathetic ganglia from T2 to T4 are effective in ES cases resistant to ß-blockers and other antiarrhythmics used in medical treatments.^[8,9]^ In addition, thoracic epidural anesthesia, spinal cord stimulation and stellate ganglion blockade (SGB) are among the neuromodulation techniques that can be applied.^[10]^ Studies show that SGB can reduce cardiac sympathetic tone and is an alternative bridge therapy in VAs.^[11]^ Considering surgical methods, SGB can be performed safely and more easily with bedside ultrasound (USG) device.

SGB can be applied with local anesthetic and/or steroid for both diagnostic and therapeutic purposes in different indication applications. In literature, local anesthetic use has been preferred in SGB in order to reduce cardiac sympathetic tone in the treatment of VA. The application of the method by adding steroid to the local anesthetic agent can increase membrane stabilization and prolong the block time, and therefore, longer-term VA can be controlled in patients with VA resistance to medical therapy.^[12]^ In our study, we preferred to apply a combination of local anesthetic and steroid in the stellate ganglion blocking method to 10 VA patients who were resistant to medical treatment and had an ICD, and we also planned to examine the 6-month follow-up results of the patients.

## 2. Materials and method

In our study, USG guided left SGB was applied to 10 VA and ES patients with ICD who were admitted to the Department of Cardiology at Çukurova University between 2020 and 2021. The 6-month results of the patients were evaluated retrospectively. ES was defined as 3 episodes of continuous VT/VF or ICD therapy for VT/VF over a 24-hour period. Demographic data, clinical and procedural outcomes of the patients were collected from electronic hospital records. Inpatient telemetry and ICD interrogation logs were reviewed for each patient. Administration of oral and intravenous (IV) anti-arrhythmic drugs was recorded for the pre- and post-SGB periods. Written informed consent was obtained from the patients for the procedures and the article. For our study, approval was taken by Çukurova University ethics committee on 16.09.2022 with the document number 125.

### 2.1. Patient selection

Patients who applied to the Cardiology Department with the diagnosis of VA or ES and did not benefit from antiarrhythmic drug therapy were selected and evaluated by a team of 2 anesthesiologists (cardiothoracic and pain specialists) and 2 cardiologists (1 of whom is an electrophysiology specialist).

All patients received standard treatment modalities based on current American College of Cardiology/American Heart Association/Heart Rhythm Society guidelines for the management of VAs.^[13]^ These methods included combinations of therapies for reversible causes (medical treatments, metabolic injuries, myocardial ischemia) beta blockers, IV anti-arrhythmic drugs, noninvasive programmed stimulation for overrate termination, and ICD programming to optimize antitachycardia pacing and minimize shocks. The number of shocks in the last 6 months of the patients who were selected for the application was checked from the ICD device. Patients with persistent VA storms despite at least 1 antiarrhythmic drug therapy with beta-blocker and catheter ablation for VT were included in the study. After the first injection, VT or VF burden and development of shock were assessed by an implantable cardioverter-defibrillator for 48 hours following the procedure.

### 2.2. SGB technique

**Figure 1.**
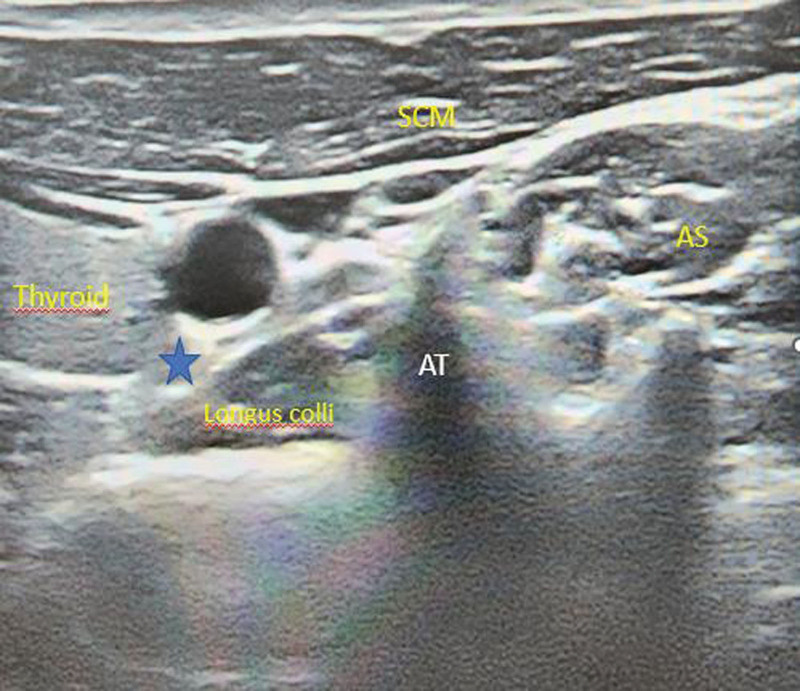
Ultrasound-guided stellate ganglion blockade. ASM = anterior scalene muscle, AT = anterior tubercule, CA = carotid artery, SCM = sternocleidomastoid muscle. Blue star: Target point.

In the operating room, the patients who were to undergo SGB were hospitalized in the supine position with the head extended. All patients with intravenous access were monitored with ECG, pulse oximetry and noninvasive blood pressure in accordance with the guidelines of the American Society of Anesthesiology. Left SGB was planned for all patients and their heads were turned to the right. The left neck and shoulder of the patient were cleaned with chlorhexidine. The linear (13-6 MHz) USG probe was prepared in accordance with the sterilization conditions. Cervical vertebra level was determined in the neck of the patient with the USG probe at the level of the cricoid cartilage. After visualizing the Chassaignac tubercle, longus coli muscle, carotid, esophagus, and thyroid gland of the C6 vertebra, an 80 mm 22-G, USG compatible (B. Braun Stimuplex Ultra 360) needle was inserted into the medial of the Chassaignac tubercle and towards the front of the prevertebral fascia of the longus colli muscle using in-plane technique (Fig. 1). After the needle site was confirmed, a negative aspiration test was performed and 10 mL of local anesthetic and steroid mixture was injected.^[14,15]^ For blockage, the solution was prepared by adding 8 mg dexamethasone, 40 mg lidocaine and 10 mg bupivacaine to 10 mL with physiological saline. The success of the procedure was evaluated with the development of Horner syndrome in the left eye. After the procedure, the patients were taken to the Cardiology Intensive Care Unit and ICD follow-up was performed for 24 hours. The medical treatments of the patients who did not develop a new VA within 24 hours were arranged and they were called for follow up check ups in the 1st and 6th months. During the check ups, the number of shocks, the presence of newly developed VA and whether the patient applied to the emergency department due to VA were followed from the ICD records.

### 2.3. Statistical analysis

Data analysis was performed using SPSS (version 11.0; SPSS Inc., Chicago, IL). The baseline variables and data were presented as counts (percentages) and continuous variables as mean ± SD. Given the non-normal distributions and small sample size, comparisons for the ICD shock numbers per 24 hours, recurrent VES at first and 6th months were made by using exact Wilcoxon Signed Rank Tests for paired analysis pre- versus post- SGB within a patient group. *P* value < .05 was considered as significant.

## 3. Results

General characteristics of the patients and cardiac and laboratory parameters before the procedure are given in Table 1. All patients included in the study had ischemic cardiomyopathy, and all of the VTs observed in the patients were monomorphic. The patients who applied to the hospital with the complaint of frequent shock despite ICD were using beta-blockers and Aldactone, while 6 patients were using ≥3 antiarrhythmic drugs. The antiarrhythmic drugs used by the patients are given in Table 2.

**Table 1:** General characteristics of the patients and cardiac and laboratory parameters before the procedure.

Age (yr)	64.37 ± 8.10	Hb (g/dL)	12.81 ± 2.15
Gender (F/M)	3/5	Wbc (10³/µL)	9.25 ± 1.10
LVEF (%)	29.87 ± 8.37	Neutrophil (10³/µL)	6.35 ± 0.97
LA (cm)	52.75 ± 7.04	Lymphocyte (10³/µL)	2.26 ± 0.87
EDD (cm)	68.62 ± 10.52	Platelet (10³/µL)	196.12 ± 56.71
ESD (cm)	55.62 ± 10.91	Uric acid (mg/dL)	6.30 ± 1.22
CK-MB (ng/mL)	8.87 ± 11.43	Na (mmol/L)	137.50 ± 3.33
Troponin (ng/L)	1474.92 ± 2728.26	Ca (mg/dL)	9.54 ± 0.52
BNP (pg/mL)	796.71 ± 584.69	K (mmol/L)	4.32 ± 0.21
HbA1c (mmol/mol)	5.87 ± 0.57	GFR (mL/dk/1.73 m²)	72.25 ± 18.36
D-dimer (mg/L)	0.8863 ± 1.07129		

BNP = brain natriuretic peptic, Ca = calcium, CK-MB = creatine kinase myocardial band, EDD = left ventricular end diastolic diameter, ESD = left ventricular end systolic pressure, F = female, GFR = glomerular filtration rate, HbA1c = Hemoglobin A 1c, Hbc = hemoglobin, K = potassium, LA = left atrial diameter, LVEF = left ventricular ejection fraction, M = male, Na = sodium, Wbc = White blood cell). All values are given as mean ± SD.

**Table 2:** Antiarrhythmic drugs used by patients.

Patient	Beta-blockers	Digoxin	Amiodarone	Mexiletine	ARNI	ACEi	Aldactone
1	Bisoprolol	−	+	+	−	+	+
2	Carvedilol	+	+	+	+	−	+
3	Bisoprolol	−	+	+	−	+	+
4	Metoprolol	−	+	+	−	+	+
5	Carvedilol	−	−	+	−	+	+
6	Bisoprolol	−	+	+	−	+	+
7	Carvedilol	−	+	−	−	+	+
8	Metoprolol	+	+	−	−	−	+

No complications developed in the patients of SGB procedure. After the procedure, the patients were followed up by being monitored for 48 hours in the Cardiology Intensive Care Unit. Resistant VA developed in 2 of 10 patients who had left SGB due to VF/VT ES and were excluded from the study. VT ablation was performed again in 2 patients who developed sustained VT on the 4th and 7th day after the SGB procedure.

**Figure 2.**
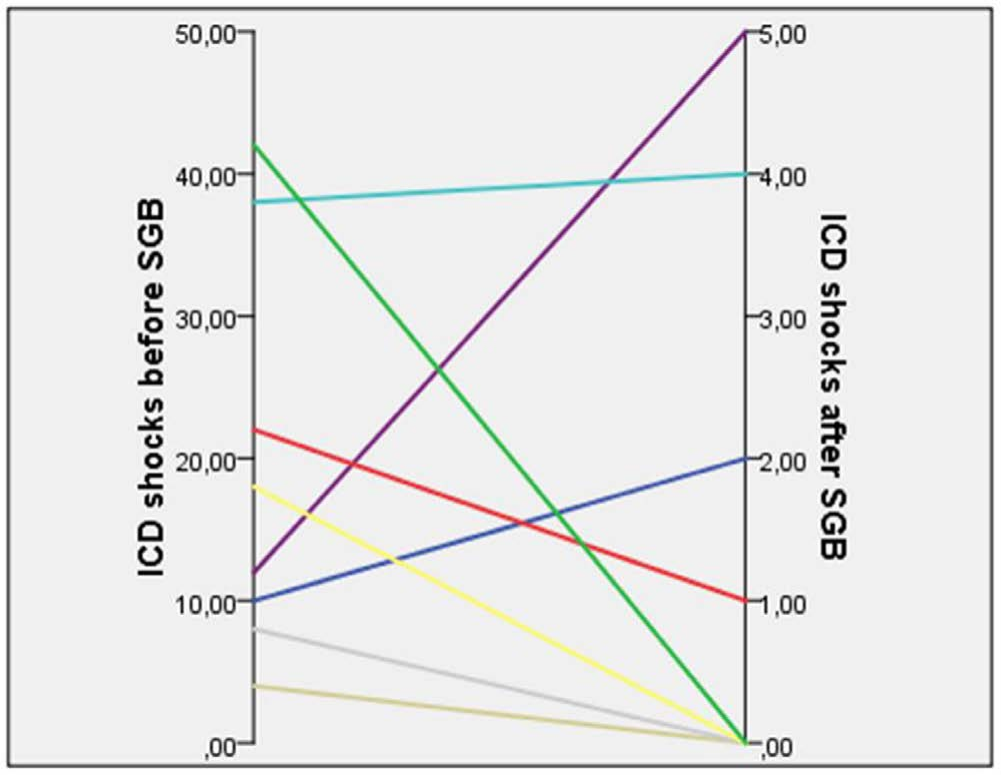
Impact of stellate ganglion block with dexamethasone on defibrillator shocks.

One (1) month after the procedure it was seen that there was a statistically significant decrease in the number of shocks in 8 patients in the 6th month controls compared to the pre-procedure (Fig. 2). The number of VES in the 1st and 6th months of the patients was also statistically significantly decreased compared to the pre-SSD (*P* = .01, *P* = .01, *P* = .01, respectively).

No additional intervention was planned for the development of VES in 3 patients at the 1st month follow-up. It was found that nonsustained VT developed in 1 patient at the 6th month follow-up, but the device did not shock because it lasted <30 seconds. In the control of the ICD, rare VES that did not require shock developed in 5 patients, while arrhythmia such as VES or VT did not develop in the other 2 patients. The number of shocks before and after SGB and development of VT/VF at 6th month are shown in Table 3. During the 6-month follow-up, no patient required hospitalization. Multiple antiarrhythmic therapy was continued in all patients.

**Table 3:** Number of shocks before and after SGB and development of VT/VF at 6th mo.

Patient	Number of shocks before SGB	Number of shocks after SGB (1st mo)	Development of VT/VF (6th mo)
1	10	2	Nonsustained VT
2	42	0	-
3	4	0	-
4	12	5	-
5	18	0	-
6	22	1	-
7	38	4	-
8	8	0	-

SGB = stellate ganglion blockade, VES = ventricular extrasystole, VF = ventricular fibrillation, VT = ventricular tachycardia.

## 4. Discussion

In our study, we observed that the local anesthetic and steroid combination, which we prefer in SGB application, in order to prolong the duration of sympathetic block in the treatment of VA resistant to medical therapy, and USG-guided method is safe. It terminates arrhythmia, and the effect continues during the 6-month follow-up period. An ES is a serious, life-threatening medical condition.^[13]^ Previous studies have shown that patients with ES have a 2 to 8 times greater risk of death in the first 3 months compared to patients without ES, and medical treatment is generally unsuccessful.^[5,16]^

In patients with ICD, ESs increase the risk of sudden death by 8 times.^[5]^ Since most patients with recurrent VA are hemodynamically unstable and require advanced hemodynamic support.^[17]^ They are often too unstable to choose sympatholytic treatments such as high-dose antiadrenergic therapy or surgical sympathectomy.^[8,18]^ SGB with USG guidance is a safe treatment method that can be applied at the bedside in the intensive care unit in hemodynamically unstable or risky patients. In the 2 studies in which SGB was applied, it was shown that the development of VA was terminated by an average of 75% after the block and it reduced the formation of VA.^[19,20]^ Fudim et al,^[11]^ showed in a meta-analysis that SGB reduced VA episodes from 16.5 to 1.40 while Lingjin et al,^[21]^ showed that it reduced it from 12.40 to 1.04. In studies with SGB activity, Tian et al,^[19]^ reported 11 ventricular arrhythmias in 72 hours while Fudim et al,^[20]^ reported 5.5 ventricular arrhythmia events in 24 hours. In our study, we compared the past 6-month records of patients with ICD and the 6-month results after SGB. In our 6-month results, we found a decrease of 77.9%.

While SGB is applied unilaterally in these studies, there are also studies showing that the bilateral application of blockade is not superior to unilateral application.^[11,19]^ Tian et al^[19]^ in their study, suggest that bilateral SGB is not superior to unilateral left SGB, and that it should be applied bilaterally in intubated patients and if ES persists. We, too, applied only left SGB in our study.

The duration of action of local anesthetics used in SGB is longer than their half-life. Tian et al^[19]^ showed that VA was suppressed in 60% of 30 patients in the first 24 hours and 50% in the first 72 hours in their SGB blocks with bupivacaine. The prolonged duration of arrhythmia suppression suggests that the overall effect of SGB involves other mechanisms than isolated suppression of efferent sympathetic nerve traffic alone.^[22–24]^ In this regard, it has been suggested that blockade of cardiac sympathetic nerves may also lead to delayed inhibition of neural or cardiac remodeling.^[25]^ It is known that steroids used as adjuvants in the treatment of chronic pain have neuronal membrane stabilization as well as their anti-inflammatory effects.^[12]^ In our study, we used dexamethasone in addition to local anesthetics in SGB application in order to prolong the blocking effectiveness and duration as a result of the additive interaction of the membrane stabilization effects of steroids and the effects of local anesthetics. We observed that it suppressed the development of VA clinically during the 6-month follow-up period.

There are many limitations in our study. In this case series, we present a retrospective analysis of prospectively collected data from cases undergoing SGB. In addition, the effect of adding steroids could not be compared, since there was no randomized controlled study and there was no control group in which only local anesthetic was administered. Procedural success, including changes in skin temperature or heart rate parameters after SGB, could not be consistently evaluated following SGB. Insufficient number of patients can be considered as a limitation to offer another suggestion. However, we believe that the results of the unilateral SGB method, which we applied with a combination of local anesthetic and steroid, will contribute to the limited literature with case series in which successful SGB was applied but the short-term effect was revealed.

In conclusion, unilateral USG-guided SGB application is an effective and safe method in patients with ES and VA. Long-term results can be satisfactory in successful responders in whom SGB is performed with a combination of local anesthetic and steroid.

## Author contributions

**Investigation:** Çağlar Özmen, Ömer Tepe.

**Methodology:** Hayri T. Özbek, Ali Deniz.

**Project administration:** Çağatay Küçükbingöz.

**Writing – review & editing:** Çağatay Küçükbingöz.

## References

[1] Pokorney SD Mi X Hammill BG. Use of antiarrhythmic medications in Medicare part D patients with an implantable cardioverter defibrillator and ventricular tachycardia. Am J Cardiol. 2017;119:1401–6. 28341360 10.1016/j.amjcard.2017.01.030

[2] Zheng ZJ Croft JB Giles WH. Sudden cardiac death in the United States, 1989 to 1998. Circulation. 2001;104:2158–63. 11684624 10.1161/hc4301.098254

[3] Aliot EM Stevenson WG Almendral Garrote JM. EHRA/HRS expert consensus on catheter ablation of ventricular arrhythmias: developed in a partnership with the European Heart Rhythm Association (EHRA), a Registered Branch of the European Society of Cardiology (ESC), and the Heart Rhythm Society (HRS); in collaboration with the American College of Cardiology (ACC) and the American Heart Association (AHA). Heart Rhythm. 2009;6:886–933. 19467519 10.1016/j.hrthm.2009.04.030

[4] Gao D Sapp JL. Electrical storm: definitions, clinical importance, and treatment. Curr Opin Cardiol. 2013;28:72–9. 23160339 10.1097/HCO.0b013e32835b59db

[5] Poole JE Johnson GW Hellkamp AS. Prognostic importance of defibrillator shocks in patients with heart failure. N Engl J Med. 2008;359:1009–17. 18768944 10.1056/NEJMoa071098 PMC2922510

[6] Zipes DP Camm AJ Borggrefe M. ACC/ AHA/ESC 2006 guidelines for management of patients with ventricular arrhythmias and the prevention of sudden cardiac death–executive summary: a report of the American College of Cardiology/American Heart Association Task Force and the European Society of Cardiology Committee for Practice Guidelines (writing committee to develop guidelines for management of patients with ventricular arrhythmias and the prevention of sudden cardiac death) Developed in collaboration with the European Heart Rhythm Association and the Heart Rhythm Society. J Am Coll Cardiol. 2006;48:1064–108. 10.1016/j.jacc.2006.07.010 16949478

[7] Waldron NH Fudim M Mathew JP. Neuromodulation for the treatment of heart rhythm disorders. JACC Basic Transl Sci. 2019;4:546–62. 31468010 10.1016/j.jacbts.2019.02.009 PMC6712352

[8] Ajijola OA Lellouche N Bourke T. Bilateral cardiac sympathetic denervation for the management of electrical storm. J Am Coll Cardiol. 2012;59:91–2. 22192676 10.1016/j.jacc.2011.09.043 PMC3470807

[9] Schwartz PJ Zaza A Locati E. Stress and sudden death. The case of the long QT syndrome. Circulation. 1991;83:II71–80. 2009631

[10] Shivkumar K Ajijola OA Anand I. Clinical neurocardiology defining the value of neuroscience-based cardiovascular therapeutics. J Physiol. 2016;594:3911–54. 27114333 10.1113/JP271870 PMC4945719

[11] Fudim M Boortz-Marx R Ganesh A. Stellate ganglion blockade for the treatment of refractory ventricular arrhythmias: a systematic review and meta-analysis. J Cardiovasc Electrophysiol. 2017;28:1460–7. 28833780 10.1111/jce.13324

[12] Manchikanti L. Role of neuraxial steroids in interventional pain management. Pain Physician. 2002;5:182–99. American Society of Interventional Pain Physicians. 16902669

[13] Al-Khatib SM Stevenson WG Ackerman MJ. 2017 AHA/ACC/HRS guideline for management of patients with ventricular arrhythmias and the prevention of sudden cardiac death: a report of the American College of Cardiology/American Heart Association Task Force on Clinical Practice Guidelines and the Heart Rhythm Society. J Am Coll Cardiol. 2018;72:e91–e220. 29097296 10.1016/j.jacc.2017.10.054

[14] Kapral S Krafft P Gosch M. Ultrasound imaging for stellate ganglion block: direct visualization of puncture site and local anesthetic spread. A pilot study. Reg Anesth. 1995;20:323–8. 7577781

[15] Narouze S. Ultrasound guided stellate ganglion block: safety and efficacy. Curr Pain Headache Rep. 2014;18:424–8. 24760493 10.1007/s11916-014-0424-5

[16] Guerra F Shkoza M Scappini L. Role of electrical storm as a mortality and morbidity risk factor and its clinical predictors: a metaanalysis. Europace 2014;16:347–53. 24096960 10.1093/europace/eut304

[17] Enriquez A Liang J Gentile J. Outcomes of rescue cardiopulmonary support for periprocedural acute hemodynamic decompensation in patients undergoing catheter ablation of electrical storm. Heart Rhythm 2018;15:75–80. 28917560 10.1016/j.hrthm.2017.09.005

[18] Wilde AA Bhuiyan ZA Crotti L. Left cardiac sympathetic denervation for catecholaminergic polymorphic ventricular tachycardia. N Engl J Med. 2008;358:2024–9. 18463378 10.1056/NEJMoa0708006

[19] Tian Y Wittwer ED Kapa S. Effective use of percutaneous stellate ganglion blockade in patients with electrical storm. Circ Arrhythm Electrophysiol. 2019;12:e007118. 31514529 10.1161/CIRCEP.118.007118

[20] Fudim MQY Waldron NH Boortz-Marx RL. Stellate ganglion blockade for the treatment of refractory ventricular arrhythmias. J Am Coll Cardiol EP. 2020;6:562–7. 10.1016/j.jacep.2019.12.017 32439042

[21] Meng L Tseng C Shivkumar K. Efficacy of stellate ganglion blockade in managing electrical storm A systematic review. JACC: Clin Electropsiol. 2017;3:942–9. 10.1016/j.jacep.2017.06.006 PMC5734652 29270467

[22] Ajijola OA Yagishita D Reddy NK. Remodeling of stellate ganglion neurons after spatially targeted myocardial infarction: neuropeptide and morphologic changes. Heart Rhythm. 2015;12:1027–35. 25640636 10.1016/j.hrthm.2015.01.045 PMC4411181

[23] Han S Kobayashi K Joung B. Electroanatomic remodeling of the left stellate ganglion after myocardial infarction. J Am Coll Cardiol. 2012;59:954–61. 22381432 10.1016/j.jacc.2011.11.030 PMC3975658

[24] Ajijola OA Hoover DB Simerly TM. Inflammation, oxidative stress, and glial cell activation characterize stellate ganglia from humans with electrical storm. JCI Insight. 2017;2:e94715. 28931760 10.1172/jci.insight.94715 PMC5621921

[25] Bradfield JS Ajijola OA Vaseghi M. Mechanisms and management of refractory ventricular arrhythmias in the age of autonomic modulation. Heart Rhythm. 2018;15:1252–60. 29454137 10.1016/j.hrthm.2018.02.015

